# Small-for-Size Liver Transplantation Increases Pulmonary Injury in Rats: Prevention by NIM811

**DOI:** 10.1155/2012/270372

**Published:** 2012-05-22

**Authors:** Qinlong Liu, Hasibur Rehman, Russell A. Harley, John J. Lemasters, Zhi Zhong

**Affiliations:** ^1^Department of Pharmaceutical and Biomedical Sciences, Medical University of South Carolina, P.O. Box 250140, Charleston, SC 29425, USA; ^2^Department of Pathology and Laboratory Medicine, Medical University of South Carolina, Charleston, SC 29425, USA; ^3^Department of Biochemistry and Molecular Biology, Medical University of South Carolina, Charleston, SC 29425, USA; ^4^Hollings Cancer Center, Medical University of South Carolina, Charleston, SC 29425, USA

## Abstract

Pulmonary complications after liver transplantation (LT) often cause mortality. This study investigated whether small-for-size LT increases acute pulmonary injury and whether NIM811 which improves small-for-size liver graft survival attenuates LT-associated lung injury. Rat livers were reduced to 50% of original size, stored in UW-solution with and without NIM811 (5 *μ*M) for 6 h, and implanted into recipients of the same or about twice the donor weight, resulting in half-size (HSG) and quarter-size grafts (QSG), respectively. Liver injury increased and regeneration was suppressed after QSG transplantation as expected. NIM811 blunted these alterations >75%. Pulmonary histological alterations were minimal at 5–18 h after LT. At 38 h, neutrophils and monocytes/macrophage infiltration, alveolar space exudation, alveolar septal thickening, oxidative/nitrosative protein adduct formation, and alveolar epithelial cell/capillary endothelial apoptosis became overt in the lungs of QSG recipients, but these alterations were mild in full-size and HSG recipients. Liver pretreatment with NIM811 markedly decreased pulmonary injury in QSG recipients. Hepatic TNF*α* and IL-1*β* mRNAs and pulmonary ICAM-1 expression were markedly higher after QSG transplantation, which were all decreased by NIM811. Together, dysfunctional small-for-size grafts produce toxic cytokines, leading to lung inflammation and injury. NIM811 decreased toxic cytokine formation, thus attenuating pulmonary injury after small-for-size LT.

## 1. Introduction

Pulmonary complications including acute lung injury and acute respiratory distress syndrome frequently occur after liver transplantation (LT) and contribute significantly to perioperative and postoperative morbidity and mortality [1–4]. The frequency of pulmonary complications is reported as high as 75% in some studies [1], and the mortality rate for acute respiratory distress syndrome reaches 50%–80% [2, 5]. Prolonged cold storage, retrieval procedures, intraoperative transfusion of plasma-containing blood products, ischemia/reperfusion- (I/R-) induced graft injury, proinflammatory cytokine and chemokine formation, leukocyte recruitment and release of neutrophil elastase, pulmonary endothelial barrier disruption, and vascular hyperpermeability possibly play critical role in the development of posttransplantation acute lung injury [1, 2, 5–8]. Since primary liver graft failure is often associated with pulmonary injury, prevention and treatment of pulmonary complications could improve the outcome of LT.

Due to severe shortage of donor organs, partial LT has increased rapidly in recent years [9–11]. In adult-to-adult living donor and split LT, small-for-size syndrome occurs when the ratio of liver graft volume is less than 30–40% of the standard liver volume of recipient [9, 12]. Such small-for-size grafts are associated with increased graft injury, inhibited liver regeneration, poor graft function, more severe posttransplantation complications, and increased mortality [9, 12]. Mechanisms of small-for-size liver graft failure remain unclear but are most likely multifactorial. Energy supply is crucial for cell survival and proliferation. Therefore, compromised energy supply could lead to liver graft injury and suppressed regeneration. Our previous studies showed that free radical production in small-for-size liver grafts leads to mitochondrial dysfunction [13–15]. Mitochondrial depolarization occurring in small-for-size liver grafts is related to opening of high conductance mitochondrial permeability transition (MPT) pores [16]. MPT pore opening collapses mitochondrial membrane potential, leading to failure of oxidative phosphorylation. NIM811, a non-immunosuppressive cyclosporine A derivative, inhibits MPT pore opening by binding to cyclophilin D, a component of the pore [17]. In small-for-size liver grafts, NIM811 protects against mitochondrial depolarization, thus decreasing injury and improving liver regeneration and functional recovery [16].

Whether small-for-size LT increases pulmonary complications remains unclear. A previous report showed that liver splitting procedures cause leukocyte recruitment in the lung tissue of donor [18]. Rapid onset of acute respiratory distress syndrome is also observed after major hepatectomy [19]. Hemodynamic alterations occurring after small-for-size LT could also lead to pulmonary complications [20, 21]. Moreover, previous studies showed that hepatic I/R promotes remote organ injury, including leukocyte infiltration and parenchymal cell damage in the lung [22]. Accordingly, this study investigated whether pulmonary complications occur after small-for-size LT and whether protection against small-for-size liver graft dysfunction by NIM811 prevents postoperative acute lung injury.

## 2. Methods

### 2.1. Liver Transplantation

Male Lewis rats (170–200 g) were used for orthotopic LT [14, 23]. Briefly, livers were explanted after flushing *in situ* with 5 mL ice-cold UW cold storage solution (Barr Laboratories, Pomona, NY) via the portal vein and removed. In ice-cold UW solution, cuffs prepared from 14-gauge *i.v.* catheters were placed over the subhepatic vena cava and the portal vein. Liver mass was reduced *ex vivo* to ~50% of original size by removing the left lateral lobe, the left portion of the median lobe, and the anterior and posterior caudate lobes after ligation with 4–0 silk suture [23]. Explants were stored in UW solution at 0-1°C for 6 h and rinsed with room-temperature-lactated Ringer's solution (Abbott Laboratories, North Chicago, IL) just prior to implantation. NIM811 (5 *μ*M, Novartis Pharma Ltd., Switzerland) was added to the storage and rinse solutions. Reduced-size liver explants were implanted into recipients of similar (170–200 g) or greater body weight (350–420 g), which results in a graft weight/standard liver weight (defined as 4% body weight) of ~50% (half-size graft, HSG) and *∼*25% (quarter-size graft, QSG), respectively. Unreduced livers were implanted into recipients of similar body weights (170–200 g) as full-size grafts (FSG). The hepatic artery and bile duct were reconstructed as described previously [14]. The ratios of graft weight/standard liver weight were not significantly different between QSG with or without NIM811 treatment (*P* > 0.1 by Students' *t*-test). All animals were given humane care in compliance with institutional guidelines using protocols approved by the Institutional Animal Care and Use Committee.

### 2.2. Serum Alanine Aminotransferase (ALT)

To access liver graft injury, serum ALT was measured from blood samples collected from the vena cava at 38 h after implantation using analytical kits from Sigma Chemical (St. Louis, MO). To adjust for graft size and recipient blood volume, serum ALT was normalized by multiplying by the recipient's standard blood volume (6.4% of body weight) and dividing by graft weight [24].

### 2.3. Pulmonary and Hepatic Histology

Under pentobarbital (50 mg/kg, *i.p*.) anesthesia at various times after implantation (5, 18, and 38 h), the lung and liver were harvested and fixed with 4% paraformaldehyde in Dulbecco's phosphate buffered saline (Invitrogen Corp. Grand Island, NY) [25, 26], imbedded in paraffin and processed for histology. In sections stained with hematoxylin and eosin (H&E), lung and liver images were acquired using a Universal Imaging Image-1/AT image acquisition and analysis system (West Chester, PA) incorporating an Axioskop 50 microscope (Carl Zeiss, Inc., Thornwood, NY) and using 20x and 10x objective lenses, respectively. Alveolar septal wall thickness was quantified by image analysis of 5 randomly selected alveolar septa per field in 10 randomly selected fields per slide using an IPlab 3.7v software (BD Biosciences, Rockville, MD). Relative alveolar septal thickness was expressed as the ratios between the average thicknesses of different transplantation groups to the sham-operation group. Liver necrosis was quantified by image analysis of 10 randomly selected fields per liver in a blinded manner using the same software and calculated by dividing the necrotic areas by the total cellular area [26].

### 2.4. Immunohistochemical Staining for 5-Bromo-2′-Deoxyuridine, Myeloperoxidase, ED1, and Intracellular Adhesion Molecule-1

To assess liver regeneration, 5-bromo-2′-deoxyuridine (BrdU, 100 mg/kg *i.p*.) was injected 1 h prior to liver harvesting to detect cells synthesizing DNA. BrdU incorporation in liver sections was determined by immunohistochemical staining as described elsewhere [27, 28]. For immunohistochemistry of leukocytes and adhesion molecules in the lung tissue, pulmonary sections were deparaffinized with xylene (Mallinckrodt Baker, Paris, Kentucky) and taken through a graded series of alcohol/water mixtures to rehydrate the tissue. To stain for myeloperoxidase (MPO), an indicator of neutrophil infiltration, lung sections were immersed in 10 mM citrate acid (pH 6), heated in microwave for antigen retrieval, and then exposed to rabbit anti-MPO polyclonal antibodies (DAKO Corp., Carpinteria, CA) at a concentration of 1 : 200 in 0.1 M phosphate buffer-0.5% Tween 20 for 30 min at room temperature followed by a 20 min incubation with peroxidase-conjugated anti-rabbit IgG_1_ antibody (DAKO Corp., Carpinteria, CA) at room temperature. 3,3′-Diaminobenzidine chromagen was then added as the peroxidase substrate. After the immunostaining procedure, a light counterstain of Meyer's hematoxylin was then applied. MPO-positive cells were counted in 10 random fields per slide in a blind manner using a 40x objective lens [29]. Immunohistochemistry of ED1, a marker of monocytes/macrophages, was performed using specific antibody (Serotek, Raleigh, NC) at a dilution of 1 : 150 for 30 min at room temperature. To stain for intracellular adhesion molecule 1 (ICAM-1) in the lungs, slides were treated in microwave as described previously for antigen retrieval and then exposed to rabbit anti-ICAM-1 polyclonal antibodies (BD Biosciences Pharmingen, San Diego, CA) at a concentration of 1 : 200 overnight at 4°C.

### 2.5. Immunoblotting

Liver tissue was homogenized in 0.1 M phosphate buffer (pH 7.2) containing 0.1% SDS, 1% IGEPal, 1% protease, and 1% phosphatase inhibitor cocktails (Sigma, St. Louis, MO) and centrifuged at 14,000 ×g for 15 min at 4°C. Aliquots of supernatant (40 *μ*g of protein) were separated on NuPAGE 4–12% Bis-Tris gels, transferred onto nitrocellulose membranes, and immunoblotted with primary antibodies specific for proliferating cell nuclear antigen (PCNA; Dako, Glostrub, Denmark) at 1 : 1000 and actin (ICN, Costa Mesa, CA) at 1 : 3000 over night at 4°C. Horseradish peroxidase-conjugated secondary antibodies were applied, and detection was by chemiluminescence (Pierce Biotec., Rockford, IL).

### 2.6. Detection of Interleukin-1*β* and Tumor Necrosis Factor-*α* mRNAs by Quantitative Real-Time PCR

Total RNA was isolated from liver tissue with Trizol (Invitrogen, Grand Island, NY). Single stranded cDNAs were synthesized from RNA (2 mg) from liver tissue using a Bio-Rad iScript cDNA Synthesis kit (Bio-Rad, Hercules, CA) [30]. The primer sequences are listed in Table 1. qPCR was conducted using a CFX96 Real-Time PCR Detection System (Bio-Rad, Hercules, CA). The abundance of mRNAs was normalized against hypoxanthine phospho-ribosyl-transferase (HPRT), a house-keeping gene, using the ΔΔ*Ct *method.

### 2.7. Statistical Analysis

Groups were compared using ANOVA plus a Student-Newman-Keuls post hoc test. Data shown are means ± S.E.M. Group sizes were 4 livers in each group for all parameters, as indicated in Section 3 and corresponding figure legends. Differences were considered significant at **P** < 0.05.

## 3. Results

### 3.1. Increased Liver Injury and Suppressed Regeneration of Small-for-Size Liver Grafts: Reversal by NIM811

**Figure 1:**
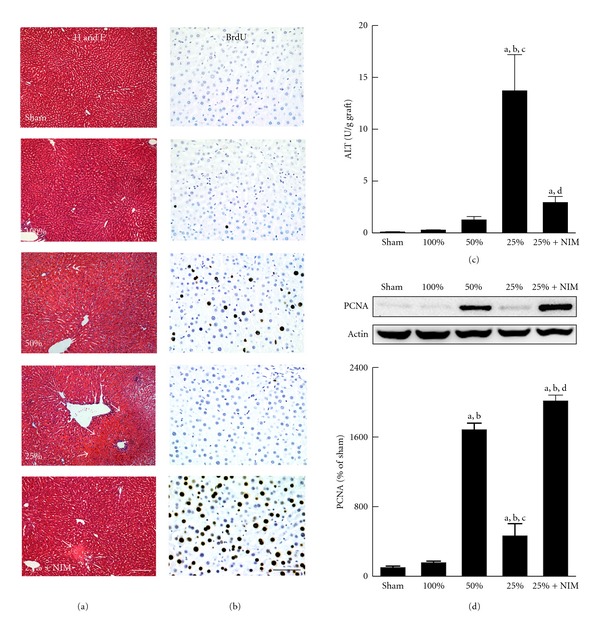
NIM811 protects against graft injury and promotes liver regeneration after transplantation of small-for-size liver grafts. Full-size and reduced-size rat livers were transplanted, as described in Section 2. In some experiments, NIM811 (NIM, 5 *μ*M) was added to the storage and poststorage lactated Ringer's rinse solutions. In (a) and (b), liver grafts were harvested at 38 h after transplantation for H&E staining ((a), bar is 100 *μ*m) or BrdU immunohistochemistry ((b), bar is 50 *μ*m). Representative images are shown. Arrows identify necrotic areas. Panels are as follow: *1st row*, liver from a sham-operated rat; *2nd row*, FSG (100%); *3rd row*, HSG (50%); *4th row,* QSG (25%); *5th row*, QSG treated with NIM811. In (c), blood samples were collected at 38 h after transplantation for ALT measurement. In (d), proliferating cell nuclear antigen (PCNA) expression in liver tissue was detected by immunoblotting and quantified by densitometry. Values are means ± S.E.M. Group sizes were 4 per group: (a) *P* < 0.05 versus sham operation; (b) *P* < 0.05 versus FSG (100%); (c) *P* < 0.05 versus HSG (50%); (d) *P* < 0.05 versus QSG (25%).

Previously, we reported that all recipients of FSG survived after transplantation [14]. Survival was decreased slightly to 80% after transplantation of HSG and markedly to 30% in QSG recipients [14]. Inhibition of the MPT by NIM811 decreased injury, improved liver regeneration, and increased survival of small-for-size liver grafts from 30% to 81% [16]. Consistent with the early work, in the present study, no pathological changes were observed in liver tissue at 5 h (data not shown) and 38 h after sham operation (Figure 1(a)), and necrosis was minimal in FSG and HSG. At 5 h after transplantation, necrosis was barely detectable in QSG (data not shown). By contrast, necrosis increased at 38 h after implantation of QSG, mainly in periportal and midzonal regions of liver lobules. NIM811 decreased necrosis in QSG (*n* = 4 per group). Serum ALT (Figure 1(c)) was *∼*0.09 U/g liver before transplantation. ALT increased at 5 h after transplantation of QSG, peaked at about 18 h, and then remained at high levels (not shown). At 38 h after implantation, ALT was *∼*14 U/g in rats receiving QSG but was only 0.3 U/g liver and 1.3 U/g liver in rats receiving FSG and HSG, respectively, indicating more severe injury in QSG. NIM811 decreased ALT to 2.9 U/g in QSG recipients (*n* = 4 per group).

Liver regeneration was evaluated by BrdU incorporation (Figure 1(b)) and expression of PCNA (Figure 1(d)). BrdU-positive cells were barely detectable at 5 h (not shown) and 38 h after sham-operation and in FSG (Figure 1(b)). In HSG, BrdU labeling was undetectable at 5 h, increased slightly after 18 h (not shown), and increased sharply at 38 h (Figure 1(b)). Proliferating cells were predominantly hepatocytes [13]. In contrast, BrdU-positive cells were rare in QSG at all time points (*n* = 4 per group). PCNA was barely detectable in sham-operated livers and FSG but increased substantially in HSG (*∼*160-fold) at 38 h, consistent with cell proliferation. PCNA expression increased only *∼*4.6-fold in QSG (*n* = 4 per group). These results show suppression of cell proliferation in QSG, which NIM811 largely reversed.

### 3.2. Pulmonary Leukocyte Infiltration and Injury Is Greater after Small-for-Size Liver Transplantation

**Figure 2:**
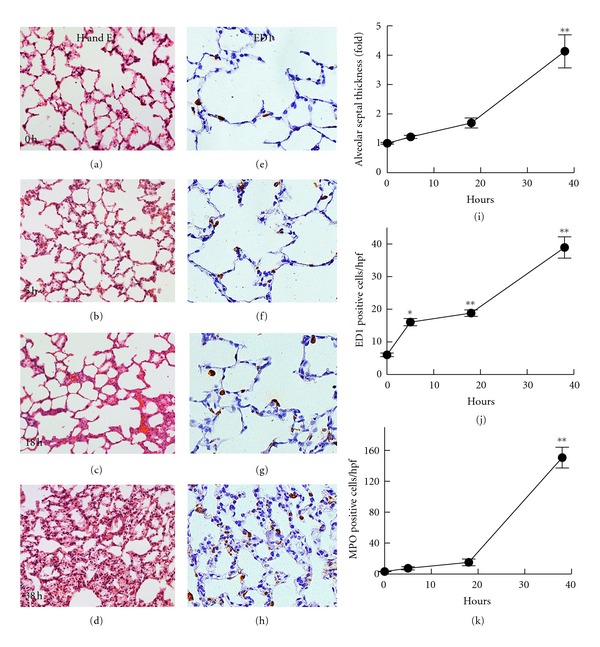
Time course of pulmonary histopathological changes after transplantation of small-for-size liver grafts. In (a)–(d), lungs were harvested at 5, 18, and 38 h after transplantation of QSG. Lung sections were stained with H&E. In (e)–(h), representative images of ED1 staining (*n* = 4 per group) are shown. In (i), alveolar septal thickness was quantified by image analysis of 5 random alveolar septa per image and 10 random images per slide using IPlab 3.7v software. Relative alveolar septal thickness was expressed as the ratio between the thicknesses of different transplantation groups to the sham-operation group. In (j) and (k), ED1 (j) and myeloperoxidase (MPO, (k)) positive cells per high power field (hpf) were counted in 10 random fields using a 40x objective lens in a blinded manner. Values are means ± S.E.M. Group sizes are 4 per group: * *P* < 0.05 and ** *P* < 0.01 versus 0 h.

Pulmonary leukocyte infiltration and injury were evaluated at 5, 18, and 38 h after LT. At 5 h after transplantation of QSG, very mild perivascular edema was observed. However, alveolar septal thickness was not significantly increased (Figures 2(b) and 2(i)). ED1-positive monocytes/macrophages increased slightly (Figures 2(f) and 2(j)), but neutrophils were not increased (Figure 2(k)). At 18 h after transplantation, the alveolar septa were slightly thickened compared to 5 h, but leukocyte infiltration remained at low levels (Figures 2(c), 2(g), 2(i), 2(j), and 2(k)). At 38 h after transplantation of QSG, alveolar septa were markedly thickened (4.1-fold) with increased cellularity (Figures 2(d) and 2(i)). Numerous leukocytes, including neutrophils and mononuclear cells, were seen in the peribonchial spaces, the lumina of blood vessels, and the perivascular as well as intra-alveolar space. At 38 h, ED1-positive cells (monocytes/macrophages) increased ~6.5-fold and MPO-positive cells (neutrophils) increased *∼*47-fold (Figures 2(j) and 2(k)) (*n* = 4 per group for all parameters). Together, these findings show that lung pathological changes became marked at *∼*38 h after transplantation of small-for-size grafts. At earlier times after QSG transplantation, infiltration was slight and composed predominantly of mononuclear cells.

**Figure 3:**
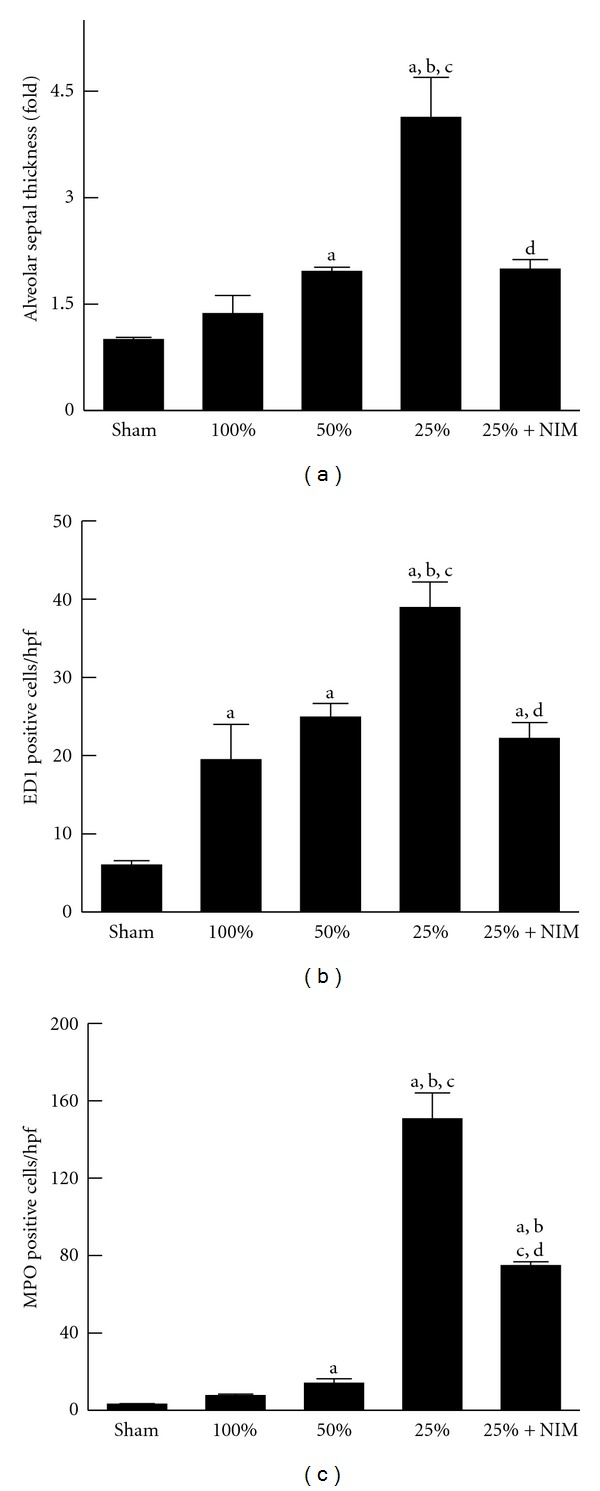
NIM811 decreases lung inflammation after small-for-size liver transplantation. Full-size and reduced-size rat livers were transplanted, as described in Section 2. Lungs were harvested 38 h after transplantation. Alveolar septal thickness (a) was quantified by image analysis of 5 random alveolar septa per image from 10 random images per H&E-stained slide using a IPlab 3.7v software. ED1 positive cells (b) and myeloperoxidase (MPO) positive cells (c) after immunohistochemical staining were counted in 10 random fields per slide using a 40x objective lens. *Sham*: lungs from sham-operated rats; 100%: lungs from FSG recipients; 50%: lungs from HSG recipients; 25%: lungs from QSG recipients; 25% + NIM: lungs from recipients of QSG treated with NIM811. Values are means ± S.E.M. Group sizes were 4 per group: (a) *P* < 0.05 versus sham operation; (b) *P* < 0.05 versus FSG (100%); (c) *P* < 0.05 versus HSG (50%); (d) *P* < 0.05 versus QSG (25%).

Lung injury was compared among groups at 38 h after transplantation. After transplantation of FSG, alveolar septa were not thickened, and neutrophils were not increased, but monocytes/macrophages increased *∼*3-fold (Figure 3). After transplantation of HSG, alveolar septa thickened slightly (1.9-fold), and monocyte/macrophages increased *∼*3-fold, but neutrophils were not increased. Overall, after transplantation of QSG, alveolar septal thickening, increased cellularity, monocyte/macrophage, and neutrophil sequestration were substantially more severe compared to recipients of FSG and HSG (Figure 3) (*n* = 4 per group).

### 3.3. NIM811 Attenuates Lung Injury after Transplantation of Small-for-Size Liver Grafts

Since NIM811 decreased hepatic injury after small-for-size LT, we examined the effects of NIM811 on lung injury. Compared to vehicle, NIM811 decreased alveolar septal thickening by 52%, monocyte/macrophage infiltration by 44% and neutrophil infiltration by 51% in QSG recipients (Figure 3) (*n* = 4 per group). Overall, alveolar septal thickening and monocyte/macrophage infiltration after NIM811 treatment of QSG returned to levels close to those observed after transplantation of FSG and HSG. Neutrophil infiltration, although diminished, remained increased relative to FSG and HSG.

### 3.4. Oxidative/Nitrosative Stress and Apoptosis after Transplantation of Small-for-Size Liver Grafts: Protection by NIM811

**Figure 4:**
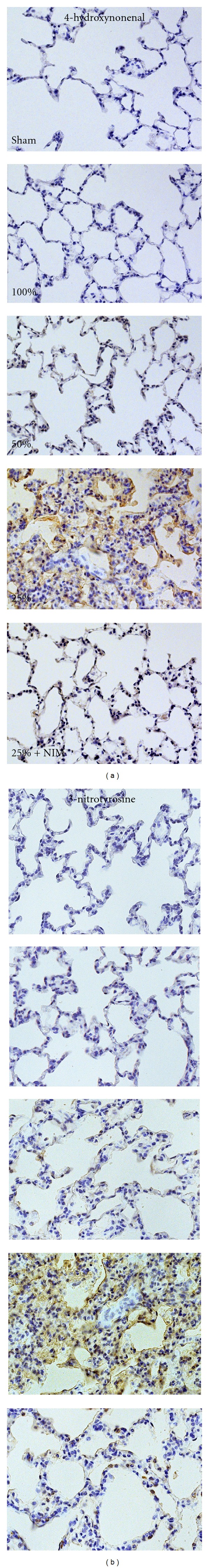
Increased pulmonary oxidative and nitrosative adduct formation after transplantation of small-for-size liver grafts: Prevention by NIM811. Lungs were harvested 38 h after transplantation. Representative images of pulmonary slides after immunohistochemical staining for 4-hydroxynonenal (*left column*) and 3-nitrotyrosine (*right column*) are shown. Panels are as follow: *1st row*, lung from a sham-operated rat; *2nd row*, lung from a FSG recipient (100%); *3rd row*, lung from a HSG recipient (50%); *4th row,* lung from a QSG recipient (25%); *5th row*, lung from a recipient of a QSG treated with NIM811. Group sizes were 4 per group.

Previous studies show that oxidative and nitrosative stresses occur in pulmonary injury after hepatic I/R [22, 31]. Infiltrating leukocytes can produce reactive oxygen and nitrogen species, leading to oxidative and nitrosative stresses and cell death in the lung. Oxidative and nitrosative stresses were evaluated by 4-hydroxynonenal and 3-nitrotyrosine adduct formation, respectively (Figure 4) (*n* = 4 per group) [32]. 4-Hydroxynonenal and 3-nitrotyrosine adducts were barely detectable in the lung tissue after sham operation and full-size LT (Figure 4). At 38 h after implantation of HSG, 4-hydroxynonenal and 3-nitrotyrosine adducts in the lung increased slightly. By contrast, 4-hydroxynonenal and 3-nitrotyrosine immunostaining became marked after transplantation of QSG (Figure 4). Staining occurred in some leukocytes, vascular endothelial cells and alveolar epithelial cells. NIM811 partially decreased hydroxynonenal and nitrotyrosine protein adducts in the lung after transplantation of QSG (Figure 4).

**Figure 5:**
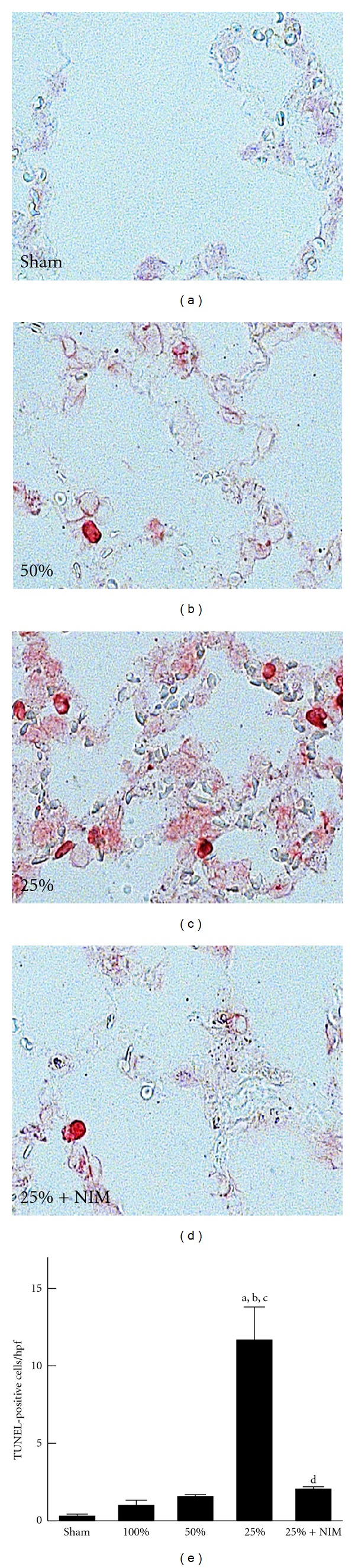
NIM811 protects against apoptosis in the lung after transplantation of small-for-size liver grafts. Lungs were harvested 38 h after transplantation. Representative images of pulmonary slides (*n* = 4 per group) after TUNEL staining are shown in (a)–(d). TUNEL-positive cells were counted in 10 random fields per slide using a 20x objective lens (e). *Sham*: lungs from sham-operated rats; 100%: lungs from FSG recipients; 50%: lungs from HSG recipient; 25%: lungs from QSG recipients; 25% + NIM: lungs from recipients of QSG treated with NIM811. Values are means ± S.E.M. Group sizes were 4 per group. (a) *P* < 0.05 versus sham operation; (b) *P* < 0.05 versus FSG (100%); (c) *P* < 0.05 versus HSG (50%); (d) *P* < 0.05 versus QSG (25%).

Pulmonary apoptosis was revealed by TUNEL staining. TUNEL-positive cells (shown by red nuclear staining) were rare in pulmonary tissue after sham operation and full-size LT. TUNEL-positive cells increased slightly after transplantation of HSG and markedly after transplantation of QSG (Figure 5). Apoptotic cells were primarily vascular endothelial cells, and/or alveolar epithelial cells. Pretreatment of liver grafts with NIM811 decreased apoptosis in the lung after transplantation of QSG (Figure 5) (*n* = 4 per group).

### 3.5. NIM811 Decreased Toxic Cytokine Formation in Small-for-Size Liver Grafts

**Figure 6:**
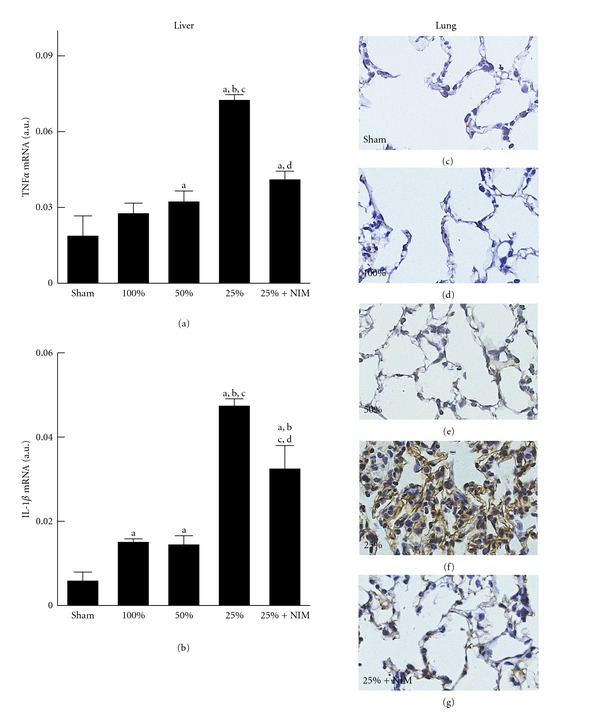
Hepatic toxic cytokine formation and pulmonary expression of adhesion molecules increased after transplantation of small-for-size liver grafts: Reversal by NIM811. Liver grafts and lungs were harvested at 38 h after transplantation. TNF*α* (a) and IL-1*β* (b) mRNAs were detected by real-time PCR. Values are means ± S.E.M. Group sizes were 4 per group. (a) *P* < 0.05 versus sham operation; (b) *P* < 0.05 versus FSG (100%); (c) *P* < 0.05 versus HSG (50%); (d) *P* < 0.05 versus QSG (25%). In (c)–(g) pulmonary ICAM-1 expression was detected immunohistochemically, and representative images are shown (*n* = 4 per group): (c) lung from a sham-operated rat; (d) lung from a FSG recipient (100%); (e) lung from HSG graft recipient (50%); (f) lung from QSG recipient (25%); (g) lung from a recipient of QSG pretreated with NIM811.

 Failing liver grafts possibly produce toxic, inflammatory cytokines, resulting in inflammation in remote organs. Accordingly, we measured hepatic cytokine expression after transplantation (*n* = 4 per group for all cytokines). Tumor necrosis factor alpha (TNF*α*) mRNA did not increase in FSG and increased only slightly (1.7-fold) in HSG (Figure 5(a)). By contrast after transplantation of QSG, TNF*α* mRNA increased ~4-fold. NIM811 treatment of QSG decreased TNF*α* mRNA by 43% (Figure 6(a)). Similar changes occurred for interleukin-1*β* (IL-1*β*). IL-1*β* mRNA increased slightly (*∼*2.5-fold) in both FSG and HSF but increased 8.1-fold in QSG (Figure 6(b)). NIM811 blunted the increase of IL-1*β* mRNA in QSG by 32%.

### 3.6. NIM811 Decreased Adhesion Molecule Expression in the Lung after Transplantation of Small-for-Size Liver Grafts

Proinflammatory cytokines released from the failing grafts may promote adhesion molecule expression in remote organs, thus promoting leukocyte infiltration and inflammation in these organs. Accordingly, we investigated intercellular adhesion molecule 1 (ICAM-1) expression in the lung (*n* = 4 per group). ICAM-1 was barely detectable in lungs of sham-operated rats and recipients of FSG (Figures 6(c) and 6(d)). ICAM-1 expression increased slightly after transplantation of HSG and substantially after transplantation of QSG (Figures 6(e) and 6(f)). ICAM-1 expression increased in vascular endothelial cells and alveolar epithelial cells (Figure 6), as well as bronchial epithelial cells (not shown). NIM811 blunted pulmonary IACM-1 expression after transplantation of QSG (Figure 6(g)).

## 4. Discussion

### 4.1. Acute Lung Injury Increases after Transplantation of Small-for-Size Liver Grafts

Pulmonary complications are severe and life-threatening conditions that adversely affect the clinical outcomes of LT, leading to high mortality [1, 3, 5]. Many factors during transplantation may cause pulmonary complications. LT often involves substantial blood loss, necessitating blood transfusion, and large fluid shifts, which can lead to pulmonary edema [1, 3, 5]. Liver I/R injury can cause damage to remote organs, such as lung and kidney [22]. Prolonged cold storage aggravates damage to donor livers and leads to acute lung injury after LT [6]. Pulmonary complications also occur frequently in patients with fulminant hepatic failure [33], suggesting interactions of liver and pulmonary functions. Clearly, prevention of pulmonary complications is crucial for increasing survival after LT.

With more frequent application of partial LT, prevention and treatment of the small-for-size syndrome become increasingly important for improving the clinical outcomes. Therefore, in this study we investigated whether transplantation of small-for-size liver grafts increases the risk of acute lung injury after transplantation. After transplantation of FSG, lung injury was minimal (Figure 3). After transplantation of HSF, pathological changes in the lung were modest (Figure 3). In contrast, transplantation of QSG resulted in overt pathological changes in the lung, including infiltration of inflammatory cells, increased alveolar septal cellularity and thickness, exudates in alveoli, oxidative/nitrosative adduct formation, and vascular endothelial/pulmonary epithelial cell death (Figures 2, 3, 4, and 5). These results indicate that small-for-size LT increases lung injury. Interestingly, lung injury occurred at a relatively late stage after transplantation (38 h), at a time point when mortality occurs [14]. These observations indicate that pulmonary complications are, at least in part, responsible for high mortality after transplantation of small-for-size liver grafts.

### 4.2. Protection of Liver Mitochondria by NIM811 Prevents Pulmonary Complications after Transplantation of Small-for-Size Liver Grafts

Lung edema could occur due to massive blood transfusion or fluid shift. Transfusion-related lung edema usually occurs early after transplantation. In this study, all recipients received similar amounts of lactated Ringer's solution during transplantation. In addition, no overt edema was detected at 5–18 h after transplantation in any groups. Therefore, the pulmonary injury observed in this study is unlikely due to transfusion. By contrast, lung injury occurred at a later stage (38 h) after transplantation and predominantly in the recipients of small-for-size liver grafts. Previous studies showed that in these small grafts, mitochondrial dysfunction occurs, leading to decreased ATP production, more severe graft injury, suppressed regeneration, and poorer liver function [13, 14, 16]. Mitochondrial dysfunction in these small-for-size grafts is not due to upregulation of uncoupling proteins but is related to onset of the MPT [16]. Opening of nonselective, highly conductive permeability transition pores in the mitochondrial inner membrane causes onset of the MPT [34]. MPT onset collapses the mitochondrial membrane potential, uncouples oxidative phosphorylation, and leads to necrotic cell death from ATP depletion [35, 37, 38]. Moreover, the MPT causes release of cytochrome *c* from the intermembrane space, which triggers activation of caspases and apoptosis [35, 36]. Growing evidence supports a critical role of the MPT in cell necrosis and apoptosis in I/R injury [37, 38]. NIM811, a MPT inhibitor, prevented hepatic mitochondrial dysfunction, decreased injury, and improved regeneration of small-for-size liver grafts [16]. This treatment increases survival after small-for-size liver grafts from 30% to 81% [16]. Here, we show that NIM811 also substantially decreases lung inflammation and injury after transplantation of QSG. Since NIM811 was added only to the cold storage solution and the poststorage rinse solution, actions of NIM811 are predominantly in the liver. Therefore, protection by NIM811 on the lung is most likely secondary to decreases in liver injury and improvement of liver graft function. A direct effect of NIM811 on lung appears unlikely, since only a small amount of NIM811 in the vasculature of quarter-size grafts enters the circulation and is distributed to other organs of the recipient. Studies will be performed in the future to determine pulmonary NIM811 concentrations at various times after transplantation of small-for-size grafts and to assess whether treatment of recipients with NIM811 at similar levels could protect against pulmonary inflammation.

### 4.3. Role of Toxic Cytokine Release from Small-for-Size Liver Grafts in Lung Injury

 How small-for-size liver grafts cause lung injury remains unclear. We tested the hypothesis that toxic cytokines released from failing small-for-size liver grafts result in lung injury. Leukocytes increased markedly in the lungs of QSG recipients but only mildly after transplantation of FSG and HSG which do not fail (Figure 3). Liver I/R injury can cause inflammatory responses in remote organs, including the lung [22]. Pulmonary neutrophil infiltration and release of elastase result in acute lung injury after LT, and this injury can be attenuated by an elastase inhibitor [39]. Prolonged cold storage increases hepatic production of proinflammatory cytokines TNF*α* and IL-1*β*, resulting in pulmonary NF*κ*-B activation and subsequent inflammatory responses and acute lung injury [6]. A previous study showed that transplantation of 60%-liver grafts does not change liver TNF*α* and IL-1*β* [25]. In the present study after transplantation of FSG, TNF*α* and IL-1*β* mRNA did not increase in the liver grafts, most likely due to the short cold storage time (Figure 6). Moreover, hepatic TNF*α* and IL-1*β* expression increased only slightly in HSG. By contrast, expression of these cytokines increased markedly in QSG (Figure 6). In parallel, pulmonary expression of ICAM-1 and leukocyte recruitment increased only modestly in the lungs of HSG recipients but substantially in QSG recipients (Figures 3 and 6). NIM811, which prevents injury to QSG, reduced hepatic TNF*α* and IL-1*β* expression, pulmonary ICAM-1 expression, and leukocyte recruitment. These results are consistent with the conclusion that failing small-for-size grafts produce toxic cytokines that promote inflammation and injury in the lung.

Surgical trauma during liver splitting also increases leukocyte sequestration into donor lungs [18]. Partial LT requires more complicated surgical procedures, and organ manipulation during liver harvest increases graft injury and activates Kupffer cells, which are the major resources of toxic cytokines in the liver [40–43]. Destruction of Kupffer cells with gadolinium chloride prevents pulmonary injury after hepatic I/R [44]. However, in this study HSG and QSG were exposed to virtually identical surgical procedures. Nonetheless, toxic cytokine formation was substantially higher in QSG than that in HSG after transplantation. Therefore, higher toxic cytokine production in QSG is unlikely to be solely due to surgical trauma. Liver injury was more severe in small-for-size liver grafts, which may stimulate inflammatory responses and toxic cytokine formation. Diminishing small-for-size graft injury by NIM811 may then decrease subsequent toxic cytokine formation.

ROS production increases markedly in small-for-size liver grafts [14]. ROS trigger opening of MPT pores [36, 45–47], and uncoupling of oxidative phosphorylation caused by the MPT further increases oxidative stress [45, 48], thus causing a vicious cycle. ROS are well known triggers of toxic cytokine formation [49, 50]. Therefore, breaking the vicious cycle of ROS production by MIN811 could also decrease toxic cytokine formation. In addition to proinflammatory cytokine production, poor liver function causes hyperbilirubinemia. Although bilirubin has antioxidant properties [51], bilirubin also causes mitochondrial toxicity and has a detrimental effect on lung surfactant surface tension properties [52–54]. Hyperbilirubinemia due to small-for-size LT may also contribute, in part, to lung injury in the recipients. It seems likely that these detrimental factors act together to promote lung injury.

## 5. Conclusions

Taken together, this study shows that acute lung injury occurs after transplantation of small-for-size liver grafts, possibly due to increased proinflammatory cytokine formation from injured and/or failing grafts with subsequent inflammatory changes in the lung. Protection of the small-for-size grafts by NIM811 diminishes this lung injury. These results also suggest that anti-inflammatory treatment can be effective in prevention of lung injury, thus improving the outcome of small-for-size LT.

## Figures and Tables

**Table 1:** Primers for Real-Time PCR.

mRNAs		Primers
IL-1*β*	Forward:	5′-AGCAGCTTTCGACAGTGAGGAGAA-3′
	Reverse:	5′-TCTCCACAGCCACAATGAGTGTGACA-3′
TNF-*α*	Forward:	5′-CAGACCCTCACACTCAGATCATCTT-3′
	Reverse:	5′-CAGAGCAATGACTCCAAAGTAGACCT-3′
HPRT	Forward:	5′-TCGAAGTGTTGGATACAGGCCAGA-3′
	Reverse:	5′-TACTGGCCACATCAACAGGACTCT-3′

IL-1*β*: interleukin-1*β*; TNF-*α*: tumor necrosis factor; HPRT: hypoxanthine phospho-ribosyl-transferase.
